# Multiple and Periodic Measurement of RBC Aggregation and ESR in Parallel Microfluidic Channels under On-Off Blood Flow Control

**DOI:** 10.3390/mi9070318

**Published:** 2018-06-24

**Authors:** Yang Jun Kang, Byung Jun Kim

**Affiliations:** 1 Department of Mechanical Engineering, Chosun University, 309 Pilmun-daero, Dong-gu, Gwangju 61452, Korea; 2 Department of Biomedical Science and Engineering, Gwangju Institute of Science and Technology (GIST), Gwangju 61005, Korea; gene392@gist.ac.kr

**Keywords:** red blood cell (RBC) aggregation, multiple microfluidic channels, master molder using xurography technique, RBC aggregation index, modified conventional erythrocyte sedimentation rate (ESR) method, regression analysis

## Abstract

Red blood cell (RBC) aggregation causes to alter hemodynamic behaviors at low flow-rate regions of post-capillary venules. Additionally, it is significantly elevated in inflammatory or pathophysiological conditions. In this study, multiple and periodic measurements of RBC aggregation and erythrocyte sedimentation rate (ESR) are suggested by sucking blood from a pipette tip into parallel microfluidic channels, and quantifying image intensity, especially through single experiment. Here, a microfluidic device was prepared from a master mold using the xurography technique rather than micro-electro-mechanical-system fabrication techniques. In order to consider variations of RBC aggregation in microfluidic channels due to continuous ESR in the conical pipette tip, two indices (aggregation index (AI) and erythrocyte-sedimentation-rate aggregation index (EAI)) are evaluated by using temporal variations of microscopic, image-based intensity. The proposed method is employed to evaluate the effect of hematocrit and dextran solution on RBC aggregation under continuous ESR in the conical pipette tip. As a result, EAI displays a significantly linear relationship with modified conventional ESR measurement obtained by quantifying time constants. In addition, EAI varies linearly within a specific concentration of dextran solution. In conclusion, the proposed method is able to measure RBC aggregation under continuous ESR in the conical pipette tip. Furthermore, the method provides multiple data of RBC aggregation and ESR through a single experiment. A future study will involve employing the proposed method to evaluate biophysical properties of blood samples collected from cardiovascular diseases.

## 1. Introduction

Normal red blood cell (RBC) in autologous plasma suspension tends to aggregate and form rouleaux (i.e., stacks-of-coins) under extremely low shear-rate conditions [1,2]. This reversible process is strongly dependent on several factors such as surface properties (membrane deformability and negative surface charge), plasma proteins (fibrinogen and globulins), shear stress, and hematocrit [3]. Additionally, RBC aggregation is considered a key determinant of blood viscosity, because it contributes to increasing blood viscosity at low shear rates. Thus, it causes to alter hemodynamic behaviors at low flow-rate regions of post-capillary venules [4,5]. Furthermore, RBC aggregation is significantly elevated in inflammatory or pathophysiological conditions [6,7]. As an indicator of RBC aggregation, erythrocyte sedimentation rate (ESR) is quantified by measuring setting distance of RBCs in a vertical tube (inner diameter = 2.55 mm, length = 300 mm, and blood volume = 5 mL) for 1 h. In other words, ESR is measured as the height of an RBC-depleted region (or plasma region) of a blood sample in a vertical tube with a specific elapse of time (*t*) (*t* = 1 h). The ESR is widely used in clinical medicine, because it is a simple and inexpensive method [6,7,8,9,10,11,12]. However, the method involves several disadvantages such as large volume consumption (~2 mL) and long measurement time (~1 h). Conversely, after blood flow in a microfluidic channel is agitated with external sources such as pressure source [13], syringe pump [2,14], and pinch valve [3], RBC aggregation is immediately quantified by constructing variations of image intensity [3,13,14,15], laser back-scattering [16], ultrasound signal [17], or electrical impedance [18] with an elapse of time (i.e., syllectogram). Previously, our group suggests the simple measurement method of ESR in the microfluidic device [15]. In other words, after setting a disposable syringe into a syringe pump, the syringe pump is reversely aligned with respect to gravitational direction. Then, blood is supplied from the top position of the syringe into a microfluidic device. Hematocrit of blood supplied into a microfluidic channel decreases over time due to continuous ESR in the disposable syringe. This variation in hematocrit is used to measure ESR by quantifying image intensity of blood within a region-of-interest (ROI) of a microfluidic channel [15]. The method is devised to monitor RBC-depleted region in a disposable syringe. However, it does not provide sufficient information on RBC aggregation of blood in a microfluidic channel. According to a previous study [17], RBC aggregation with microscopic, image-based intensity gave comparable value to RBC aggregation, compared with the ultrasonic method and conventional ESR methods. Recently, a simple method is devised to measure RBC aggregation under continuous ESR in a conical pipette tip [19]. According to the previous study [20], blood storage time at 25 °C should be limited to four hours for RBCs aggregation. Above four hours, RBC aggregation was varied with an elapse of time. In other words, when RBCs aggregation was varied over time, the repetitive test increased the scattering of the aggregation index significantly. Thus, multiple measurements of RBC aggregation were required to avoid large scattering due to repetitive tests being conducted for a long period of time. Thus, it is effective to obtain several data points without repetitive tests. Specifically, RBC aggregation should be quantified as mean ± standard deviation in single experiment with respect to an elapse of time. Thus, it does not require repetitive tests, which cause rheological properties to vary continuously.

In this study, multiple measurements of RBC aggregation under continuous ESR are proposed by sucking blood from a pipette tip into parallel microfluidic channels and quantifying image intensity, especially throughout a single experiment. Furthermore, two indices (aggregation index (AI), and erythrocyte-sedimentation-rate aggregation index (EAI) [19]) are evaluated to consider variations in RBC aggregation due to continuous ESR in a conical pipette tip. The proposed method is demonstrated by using a microfluidic device that is composed of an inlet port, an outlet port, and identical-parallel-microfluidic channels. The microfluidic device is prepared from master mold using xurography technique rather than MEMS (micro-electro-mechanical-system) fabrication techniques. A pipette tip is fitted into the inlet port, and the outlet port is then connected to a disposable syringe with a polyethylene tube. A disposable syringe is installed into a syringe pump. The syringe pump is set to a constant flow rate and operates in the withdrawal mode. A pinch valve is installed between the outlet port and a disposable syringe to control blood delivery from the pipette tip to a microfluidic device. The blood flow stops or runs in parallel microfluidic channels when the pinch valve is periodically clamped or released. The image intensity of blood in each microfluidic channel is quantified to obtain several data points of RBC aggregation over an interval of time. Thus, the proposed method measures several data points of RBC aggregation under continuous ESR in a single experiment without requiring additional repetitive tests.

The feasibility of the proposed method is evaluated by conducting experimental tests with two RBC aggregation indices such as EAI and AI. The EAI is applied to quantify RBC aggregation under continuous ESR in conical pipette tip. Furthermore, AI as a conventional aggregation index is used to compare EAI. In order to evaluate the performance of the proposed method, AI and EAI are quantified for several blood samples (hematocrit = 20%, 30%, 40%, and 50%) that are prepared by adding normal RBCs into autologous plasma. In order to elevate RBC aggregation, blood samples are prepared using normal RBCs with three different concentrations of dextran solution (*C*_dextran_ = 5 mg/mL, 15 mg/mL, and 20 mg/mL). Subsequently, the proposed method is employed to quantify the effect of dextran solution on the RBC aggregation under continuous ESR in the pipette tip.

## 2. Materials and Methods

### 2.1. Blood Sample Preparation

Blood samples were prepared by adding human RBCs to autologous plasma to evaluate RBC aggregation and ESR. Concentrated RBCs and plasma were provided by the Gwangju-Chonnam Blood Bank (Gwangju, Korea). In blood bank, RBCs were collected by removing plasma from whole blood. Then, the RBCs were stored in anticoagulant citrate phosphate dextrose adenine solution (CPDA-1). After concentrated RBCs packed within blood bag (~320 mL) were provided by blood bank, they were immediately stored at 4 °C. To collect pure RBCs from the concentrated RBCs, blood sample was prepared by adding concentrated RBCs (~8 mL) into 1× phosphate-buffered saline (PBS) solution (~8 mL). By operating centrifugal separator, pure RBCs were collected by removing buffy layer and PBS from the blood sample. This washing procedure was repeated three times. When blood storage time at 25 °C was over four hours, RBC aggregation was varied with an elapse of time [20]. Thus, after blood samples were prepared, they were stored at 4 °C before blood test. All experiments were finished within four hours. According to the previous studies [21,22], blood biophysical properties including blood viscosity, and RBC aggregation remained constant for up to 7 days of storage time. In other words, when storage time of RBCs was over 7 days, the RBCs were removed. Hematocrits of normal blood (*H*_ct_ = 20%, 30%, 40%, and 50%) were adjusted by adding normal RBCs to autologous plasma. In order to elevate RBC aggregation in blood samples, three different concentrations of dextran solution (*C*_dextran_) (*C*_dextran_ = 5 mg/mL, 15 mg/mL, and 20 mg/mL) were prepared by adding dextran (Leuconostoc spp., MW = 450–650 kDa, Sigma-Aldrich, St. Louis, MO, USA) to a 1× PBS solution. Hematocrit of blood samples was adjusted to 30% by adding normal RBCs into a specific dextran solution. A control blood sample (*C*_dextran_ = 0) was prepared by adding normal RBCs into a 1× PBS solution.

### 2.2. Fabrication of a Microfluidic Device and Experimental Procedure

**Figure 1:**
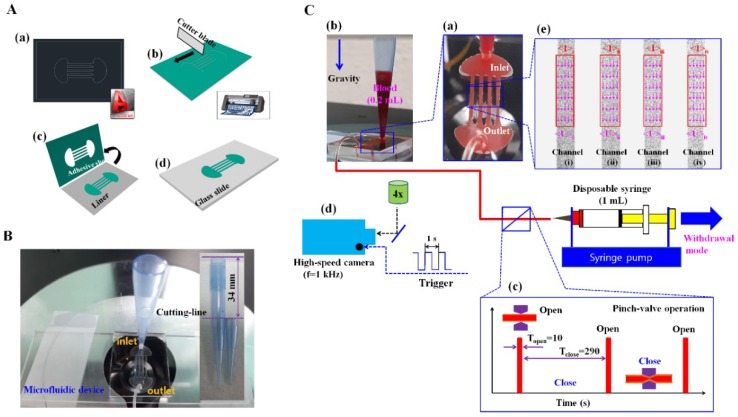
Details of a master mold using an adhesive sheet and a conical pipette tip, and schematic diagram of a proposed method for quantifying red blood cells (RBCs) aggregation and erythrocyte sedimentation rate (ESR) in a pipette tip. (**A**) Fabrication procedure for preparing an adhesive sheet for a master mold. (**A-a**) A pattern of master mold designed with commercial software. (**A-b**) A cutter blade to cut a cover of the adhesive sheet. (**A-c**) The cover was peeled off from a liner. (**A-d**) The master mold was finally prepared by attaching the liner on a glass slide. (**B**) A pipette tip tightly fitted into a microfluidic device. The pipette tip was prepared by cutting 34 mm from the top surface. (**C**) A schematic diagram of the proposed method including a pipette tip, a disposable microfluidic device, a pinch value, and a syringe pump. (**C-a**) A microfluidic device is composed of an inlet port, an outlet port, and parallel microfluidic channels (*N* = 4, width = 600 µm, depth = 100 µm, and length = 8 mm). (**C-b**) A pipette tip fitted into inlet port is filled with 0.2 mL blood. The outlet port is connected to a disposable syringe (1 mL) with a polyethylene tube (inner diameter = 250 µm, length = 600 mm). A disposable syringe is installed into the syringe pump. Flow rate is set to 2 mL/h (i.e., *Q* = 2 mL/h) at the withdrawal mode. (**C-c**) A pinch valve was installed in front of the disposable syringe to control blood flow in parallel microfluidic channels. A pinch valve was manually opened for 10 s (*T*_open_ = 10 s) and closed for 290 s (*T*_close_ = 290 s) during a single period (*T* = 300 s). (**C-d**) Image acquisition system including microscope, high-speed camera, and external trigger. (**C-e**) A specific region-of-interest (ROI) (80 pixel × 400 pixel) for each microfluidic channel was selected to determine average velocity (<*U*>_i_, <*U*>_ii_, <*U*>_iii_ and <*U*>_iv_) and average image intensity (<*I*>_i_, <*I*>_ii_, <*I*>_iii_ and <*I*>_iv_), which were calculated by conducting time-resolved micro-particle image velocimetry (PIV) technique and digital image processing, respectively.

As shown in Figure 1A, a master mold was fabricated using a cutting plotter (i.e., xurography technique) [23,24,25,26]. Here, an adhesive sheet with a thickness of 100 µm was employed for 100 μm depth. A master mold was designed by using a commercial software program (AutoCAD 2014, Autodesk, San Rafael, CA, USA) [Figure 1A-a]. A cutter blade (CE6000-40, Graphtec, Irvine, CA, USA) was used to cut a cover of the adhesive sheet [Figure 1A-b]. The cover was peeled off from a liner [Figure 1A-c]. The master mold was finally prepared by attaching the liner on a glass slide [Figure 1A-d]. PDMS (polydimethylsiloxane) was mixed at a ratio of 10:1 with a curing agent, and the mixture was poured on the master mold positioned in a Petri dish. The air bubbles dissolved in the PDMS were completely removed by operating a vacuum pump for 1 h. The PDMS was cured in a convection oven at temperature of 75 °C for 1.5 h, and the PDMS block was peeled off from the master mold. Two biopsy punches were used, and the inlet port and outlet port was displayed through holes with diameters of 1.5 mm and 0.75 mm, respectively. Following the simultaneous treatment of oxygen plasma (CUTE-MPR, Femto Science Co., Gyeonggi, Korea) on the PDMS block and on a glass substrate, the microfluidic device was prepared by bonding the PDMS block to the glass substrate.

A microfluidic device was mounted on an optical microscope (IX53, Olympus, Tokyo, Japan) equipped with a 4× objective lens (numerical aperture (NA) = 0.1). As shown in Figure 1B, a pipette tip that was tightly fitted into inlet port after a pipette tip (50–1000 µL, Eppendorf, Germany) was cut approximately 34 mm from the top surface. The outlet port was connected to a disposable syringe with a polyethylene tube. The flow rate was set to 2 mL/h (i.e., *Q* = 2 mL/h) at the withdrawal mode. Blood (0.2 mL) was dropped into the pipette tip with a pipette. In order to avoid non-specific binding of plasma protein, microfluidic channels and the pipette tip were filled with 1% bovine serum albumin (BSA) diluted with 1× PBS solution (pH 7.4, GIBCO, Life Technologies, Gangnam-gu, Korea) for 20 min. The BSA solution was removed from the microfluidic device by releasing the pinch valve. Subsequently, 0.2 mL of blood was dropped into the pipette tip, and it was ready to measure RBC aggregation under continuous hematocrit variations. As shown in Figure 1C-d, a high-speed camera (FASTCAM MINI, Photron, San Jose, CA, USA) was employed to capture blood flows in the microfluidic channels. The spatial resolution of the camera corresponded to 1280 × 1000 pixels. Each pixel corresponds to 10 µm. A function generator (WF1944B, NF Corporation, Tokyo, Japan) triggered the high-speed camera at an interval of 1 s to sequentially capture two microscopic images at a frame rate of 1 kHz. All experiments were conducted at a room temperature of 25 °C.

### 2.3. The Proposed Method for Quantifying RBCs Aggregation and ESR over Time

A simple measurement technique of RBC aggregation under continuous ESR is proposed by sucking blood from a pipette tip into parallel microfluidic channels, and quantifying image intensity of blood over an interval of time. Several data of RBC aggregation are obtained throughout a single experiment at an interval of specific time duration.

Figure 1C shows a schematic diagram of the proposed method including a disposable microfluidic device, a pipette tip, a pinch valve, and a syringe pump. A microfluidic device is designed with an inlet port, an outlet port, and parallel microfluidic channels (*N* = 4, width = 600 µm, depth = 100 µm, and length = 8 mm) [Figure 1C-a]. A pipette tip (50–1000 µL, Eppendorf, Hamburg, Germany) is cut approximately 34 mm from the top surface [Figure 1B], and the pipette tip is tightly fitted into an inlet port (inner diameter = 1.5 mm). The outlet port is connected to a disposable syringe (1 mL) with a polyethylene tube (inner diameter = 250 µm and length = 600 mm). After the disposable syringe is installed into a syringe pump, the flow rate was set to 2 mL/h (*Q* = 2 mL/h) at the withdrawal mode. Subsequently, 0.2 mL blood is dropped into the pipette tip with a pipette [Figure 1C-b]. The RBC migrates towards the button position due to continuous ESR in the pipette tip, and thus the previous method quantifies ESR by measuring the height of the RBC-depleted region [15,17]. In contrast to previous ESR measurement, the proposed method is suggested to measure variations of RBC aggregation under continuous ESR by evaluating the microscopic image-based intensity of blood flow in microfluidic channels. Therefore, to evaluate variations in hematocrit due to continuous ESR in the pipette tip, blood is supplied from the conical pipette tip to the microfluidic channels over an interval of time. A pinch valve (Supa clip, Pankyo, Gyeonggi-do, Korea) was installed between the outlet port and the disposable syringe to periodically ensure blood delivery into the microfluidic device. A pinch valve is manually opened for 10 s (*T*_open_ = 10 s) and closed for 290 s (*T*_close_ = 290 s) during a single period (*T* = 300 s) [Figure 1C-c]. As shown in Figure 1C-e, a specific ROI (80 × 400 pixels) is selected for each microfluidic channel to determine an average value of blood velocity for each microfluidic channel (<*U*>_i_, <*U*>_ii_, <*U*>_iii_ and <*U*>_iv_) and an average value of image intensity for each microfluidic channel (<*I*>_i_, <*I*>_ii_, <*I*>_iii_ and <*I*>_iv_) that are calculated by conducting time-resolved micro-PIV technique and digital image processing, respectively.

**Figure 2:**
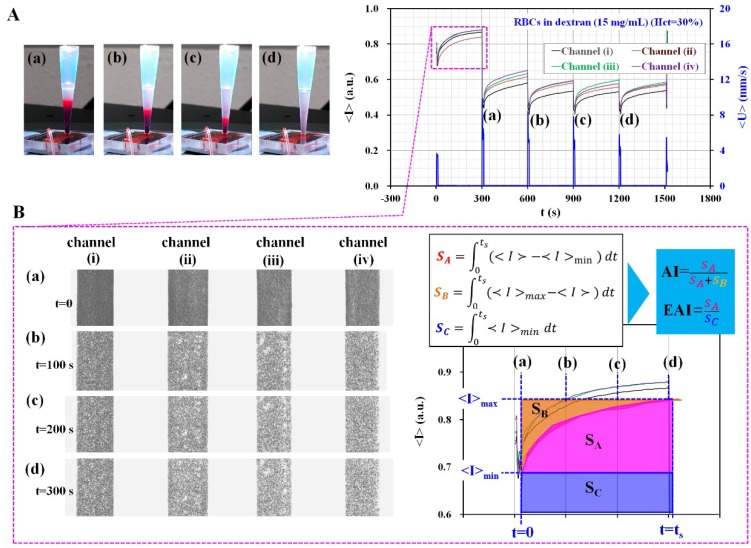
Quantification of two indices (AI and EAI) for quantifying RBCs aggregation and ESR in a conical pipette tip. (**A**) Temporal variations in <*I*> for each microfluidic channel and average velocity (<*U*>) through four microfluidic channels. Sequential images for representing RBC-depleted regions in a pipette tip with an elapse of time (*t*) ([**A-a**] *t* = 300 s, [**A-b**] *t* = 600 s, [**A-c**] *t* = 900 s, and [**A-d**] *t* = 1200 s). (**B**) Temporal variations of <*I*> for each microfluidic channel over a single period. Sequential microscopic images represent RBC aggregation for each microfluidic channel with an elapse of time (t) ([**B-a**] *t* = 0, [**B-b**] *t* = 100 s, [**B-c**] *t* = 200 s, and [**B-d**] *t* = 300 s). Two indices (AI and EAI) were defined as AI = *S*_A_/(*S*_A_ + *S*_B_) and EAI = *S*_A_/*S*_C_ from a syllectogram obtained for 300 s.

As a preliminary study, blood with 30% hematocrit was prepared by adding normal RBCs into a specific dextran solution (i.e., *C*_dextran_ = 15 mg/mL). Figure 2A shows temporal variations of averaged image intensity (<*I*>) for individual microfluidic channel and averages blood velocity (<*U*>) through four microfluidic channels. Here, to monitor temporal variations of blood flow depending on the operation of the pinch value (i.e., open or close), velocity fields were obtained by conducting time-resolved micro-PIV technique. After guaranteeing the operation of the pinch valve sufficiently, we did not try to get velocity information. Sequential images for representing RBC-depleted regions in a pipette tip were captured with elapses of time (t) ([a] *t* = 300 s, [b] *t* = 600 s, [c] *t* = 900 s, and [d] *t* = 1200 s). With respect to an elapse of time, the RBC-depleted region tended to increase due to continuous ESR in conical tip. Additionally, image intensity (<*I*>) tended to decrease due to an increase in the hematocrit. As shown in Figure 2B, temporal variations of <*I*> for each microfluidic channel are obtained for a single period (*T* = 300 s). Temporal variations of image intensity (<*I*>), which are called a syllectogram, are used to calculate three representative factors (*S*_A_, *S*_B_, and *S***_C_**) [19] as follows:

(1) $$S_{A}=\int_{t=0}^{t=t_{s}}(<I>-<I{>}_{\min})dt$$

(2) $$S_{B}=\int_{t=0}^{t=t_{s}}(<I{>}_{\max}-<I>)dt$$

(3) $$S_{C}=\int_{t=0}^{t=t_{s}}<I{>}_{\min}dt$$

In Equations (1)–(3), <*I*>_min_ and <*I*>_max_ are denoted as <*I*>_min_ = <*I* (*t* = 0)> and <*I*>_max_ = <*I* (*t* = *t*_s_)>, respectively. Furthermore, <*I*>_min_ tended to decrease due to ESR in the pipette tip, and thus S_C_ was used to represent the dynamic behavior of ESR. According to most previous methods, a blood sample was directly dropped into an inlet port of the microfluidic device. Since hematocrit of the blood sample remained constant in a microfluidic channel, the previous methods did not require to consider the effect of continuous ESR in the reservoir on the RBCs aggregation. In other words, the previous method did not consider the effect of hematocrit variations on the syllectogram (i.e., *S*_C_ = 0). In other words, as shown in right panel of Figure 2B, RBC aggregation was quantified by calculating AI as AI = *S*_A_/(*S*_A_ + *S*_B_). However, in this study, the proposed method involved simultaneously measuring RBC aggregation and ESR in conical pipette tip. The continuous ESR caused to increase hematocrit of blood supplied into a microfluidic channel. According to temporal variations of <*I*>, <*I*>_min_ decreased due to increases in hematocrit. In order to quantify RBC aggregation due to ESR in the conical pipette tip, it was necessary to simultaneously consider variations in <*I*> due to RBC aggregation (i.e., *S*_A_) and <*I*>_min_ due to ESR (i.e., *S*_C_). Thus, EAI is evaluated as EAI = *S*_A_/*S*_C_. For convenience, *t*_s_ was fixed as *t*_s_ = 250 s. Sequential microscopic images for four microfluidic channels indicated that RBC tended to gradually aggregate with respect to an elapse of time (t) ([a] *t* = 0, [b] *t* = 100 s, [c] *t* = 200 s, and [d] *t* = 300 s). This preliminary result indicated that the proposed method measured variations of RBC aggregation under continuous ESR by quantifying image intensity of blood in each microfluidic channel.

### 2.4. Quantifications of Image Intensity (<I>) and Blood Velocity (<U>)

In order to evaluate variations in image intensity of blood with an elapse of time, a specific ROI (80 pixel × 400 pixel) was selected within each microfluidic channel as shown in Figure 1C-e. Average pixel values over the ROI (<*I*>_i_, <*I*>_ii_, <*I*>_iii_, and <*I*>_iv_) were estimated by performing digital image processing with a commercial software program (Matlab, Mathworks, Natick, MA, USA). To evaluate temporal variations of blood flow, velocity fields were obtained by conducting a time-resolved micro-PIV technique to evaluate temporal variations in blood flow. Specific ROIs (80 pixel × 400 pixel) were selected for each microfluidic channel to obtain velocity fields of blood flow. The size of the interrogation window corresponded to 16 × 16 pixels. The window overlap corresponded to 50%. The obtained velocity fields were validated with a median filter. The average velocities of blood flow for each microfluidic channel (<*U*>_i_, <*U*>_ii_, <*U*>_iii_, and <*U*>_iv_) were calculated as an arithmetic average over the ROI. Finally, average blood velocity in the parallel microfluidic channel (<*U*>) was calculated as <*U*> = (<*U*>_i_ + <*U*>_ii_ + <*U*>_iii_ + <*U*>_iv_)/4.

## 3. Results and Discussion

### 3.1. Variation of Width in Microfluidic Channel Fabricated by Using an Adhesive Sheet for Master Mold

**Figure 3:**
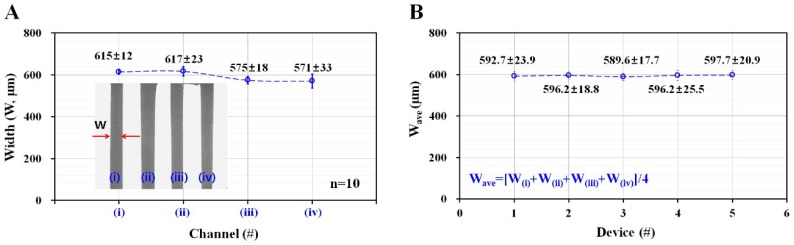
Quantitative measurement of width variations of four microfluidic channels fabricated by using an adhesive sheet for master mold. (**A**) Variations of channel width for four individual channels. (**B**) Variations of averaged channel width for five different devices.

In this study, the use of a liner cut from an adhesive sheet to form the master mold was employed to simplify the fabrication process, compared to MEMS fabrication. Variations of channel width were measured by analyzing microscopic images obtained from high-speed camera. After then, digital image processing was conducted to measure channel width of individual channel. Measurement for each channel was repeated ten times (*n* = 10). As shown in Figure 3A, the corresponding channel width (*W*) for each channel was measured as (a) *W* = 615 ± 12 µm for (i) channel, (b) *W* = 615 ± 23 µm for (ii) channel, (c) *W* = 575 ± 18 µm for (iii) channel, and (d) *W* = 571 ± 33 µm for (iv) channel. In other words, this simple technique could be employed to prepare microfluidic channel within 4% normal deviation in channel width of individual channel. Compared with MEMS fabrication, this simple technique was not feasible to fabricate a microfluidic channel with highly consistent dimensions. According to the previous studies, this xurography technique had poor resolution for dimensions smaller than 500 μm. However, it provides several advantages including inexpensive cost (material and equipment) and rapid fabrication without cleanroom [24,26]. Due to high advantages, it had been employed to fabricate biomedical device for blood flow analysis [25,27]. However, in this study, dynamic blood flow was required to disaggregate aggregated RBCs sufficiently. Since RBCs aggregation was quantified at stationary blood flows by clamping polyethylene tube with a pinch valve, blood flow in each channel does not have an influence on measurement of RBCs aggregation. Even though this technique has a little deviations in channel width, an adhesive sheet could be simply used as a master mold to prepare microfluidic device. On the other hand, to verify variations of each microfluidic device, channel width was measured with five microfluidic devices. Averaged channel width for each device was used to quantify device-to-device variation. Four channel widths of each device were arithmetically averaged as *W*_ave_ = [*W*_(i)_ + *W*_(ii)_ + *W*_(iii)_ + *W*_(iv)_]/4. As shown in Figure 3B, averaged channel width (*W*_ave_) remained consistent for five different devices. From these measurement results, the use of a liner cut from an adhesive sheet contributes to simplifying the fabrication process, and to preparing PDMS microfluidic channel reliably.

### 3.2. Quantitative Evaluation of the Effects of Pinch-Valve Operation and Syringe Pump Flow-Rate

**Figure 4:**
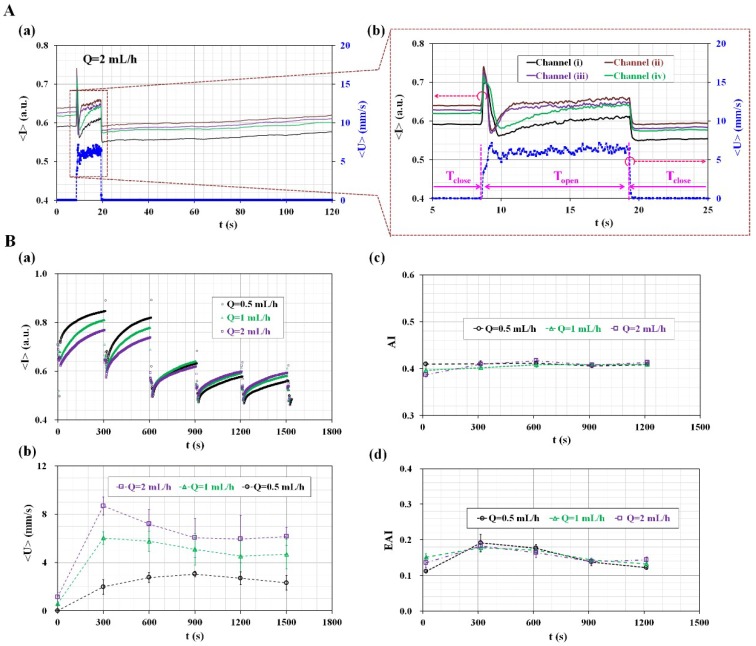
The quantitative evaluation of the effects of vital two factors including pinch-valve operation and syringe pump flow-rate on the measurement of RBC aggregation and ESR. (**A**) Dynamic characterization of image intensity (<*I*>) and blood velocity (<*U*>) based on operation of the pinch valve (open, close). To evaluate transient response with a high resolution, <*I*> and <*U*> were obtained at intervals of 0.1 s. Hematocrit of blood was adjusted to 30% by adding normal RBCs into the plasma solution. At the constant flow rate of 2 mL/h with syringe pump, temporal variations of <*I*> and <*U*> were obtained for specific duration of (**A-a**) 120 s and (**A-b**) 25 s. As a result, blood flow was stopped immediately within 0.2 s after clamping a tube with a pinch (i.e., close). (**B**) The effect of syringe pump flow-rate (*Q*) on the measurement of RBC aggregation (i.e., AI) and ESR (i.e., EAI). Temporal variations of <*I*> and <*U*> were obtained by varying flow rate of syringe pump (*Q*) (*Q* = 0.5, 1, and 2 mL/h). (**B-a**) Temporal variations of <*I*> with respect to blood flow rate. (**B-b**) Temporal variations of <*U*> with respect to syringe pump flow-rate. (**B-c**) Temporal variations of AI with respect to syringe pump flow-rate. (**B-d**) Temporal variations of EAI with respect to syringe pump flow-rate.

In order to analyze the dynamic effect of a microfluidic system (polyethylene tube and a microfluidic device) on the performance of the proposed method, image intensity (<*I*>) and blood velocity (<*U*>) of blood flow in parallel microfluidic channels were quantified with high resolution. Two sequential microscopic images were captured and recorded at an interval of 0.1 s. Hematocrit of blood was adjusted to 30% by adding normal RBCs into autologous plasma. A flow rate was set to 2 mL/h with a syringe pump. Figure 4A-a,A-b show temporal variations of image intensity (<*I*>) and average of blood velocity (<*U*>) obtained for specific durations of 120 s and 25 s. When a pinch valve clamps or releases the polyethylene tube, <*I*> and <*U*> showed transient behaviors with respect to time. The experimental results indicated that blood flow stopped shortly within 0.2 s. After clamping the tube, image intensity (<*I*>) tended to increase immediately. According to previous studies [17,28], blood flow decreased gradually based on the compliance effect of the microfluidic system when a syringe pump was used to control blood flow in a microfluidic channel in the delivery mode. In other words, when the syringe pump was set to zero value of the flow rate (i.e., *Q* = 0), RBC aggregation did not occur due to transient blood flow in the microfluidic channel. Thus, RBC aggregation was quantified by using temporal variations in image intensity after an elapse of 20 s [28] or 60 s [17]. For this reason, a glass capillary tube [2] or detour channel [14] was applied to minimize time delay due to the compliance effect of the microfluidic system. However, in the proposed method, a syringe pump sets to constant flow rate as withdrawal mode. The pinch valve clamped or released the polyethylene tube, and thus the blood flow stopped or ran within a very short time. The experimental results indicated that RBC aggregation was measured immediately after clamping the tube with a pinch valve (i.e., close mode). Additionally, the compliance effect of fluidic system on RBC aggregation and ESR was negligible.

To verify the effect of syringe pump flow-rate on RBC aggregation (i.e., AI) and ESR (i.e., EAI), temporal variations of image intensity (<*I*>) and velocity (<*U*>) were obtained by varying flow rate of the syringe pump (*Q*) (*Q* = 0.5 mL/h, 1 mL/h, and 2 mL/h). Figure 4B-a shows temporal variations of <*I*> with respect to flow rate of syringe pump (*Q*). Here, <*I*> denotes average values of image intensity in four microfluidic channels. With increasing syringe pump flow-rate, <*I*> tends to decrease. For up to 600 s, minimum value of <*I*> (i.e., <*I*>_min_) tends to decrease gradually because of continuous ESR in the conical pipette tip. After 600 s, <*I*> remained constant without respect to syringe pump flow-rate. In addition, amplitude of <*I*> (i.e., *ΔI*) was decreased over time. Figure 4B-b shows temporal variations of <*U*> with respect to syringe pump flow-rate (*Q*). Here, <*U*> denotes averaged blood velocity through four microfluidic channels, especially for 10 s per each period. By increasing flow rate, blood velocity (<*U*>) tends to increase distinctively. In addition, at the higher syringe pump flow-rate of *Q* = 1~2 mL/h, blood velocity was decreased gradually from *t* = 300 s to *t* = 900 s. Then (*t* > 900 s), blood velocity remained constant over time. However, at the lower syringe pump flow-rate of *Q* = 0.5 mL/h, blood velocity remained constant over time. Using temporal variations of <*U*> during a specific time duration of 1500 s, temporal variations of two indices (AI and EAI) were obtained by varying flow rates. As shown in Figure 4B-c,B-d, AI as conventional RBC aggregation index remained constant without respect to flow rate but not with respect to the initial condition (*t* = 0). However, due to continuous ESR in the conical pipette tip, hematocrit of blood supplied from the conical tip into microfluidic channels was increased. Thus, EAI was decreased gradually over time. In addition, syringe pump flow-rate does not contribute to varying EAI. From these experimental demonstrations, it is found that syringe pump blood-flow rate does not have a significant influence on measurement of AI and EAI. In other words, the syringe pump flow-rate of *Q* = 0.5 mL/h is considered sufficient for removing and filling blood samples in each microfluidic channel for each period. In this study, for convenience, the higher syringe pump flow-rate of *Q* = 2 mL/h was selected to easily suck and remove blood samples from the bottom area of the pipette tip and change blood samples in each microfluidic channel.

### 3.3. Quantitative Evaluation of the Channel Number for Evaluating Variations of AI and EAI

**Figure 5:**
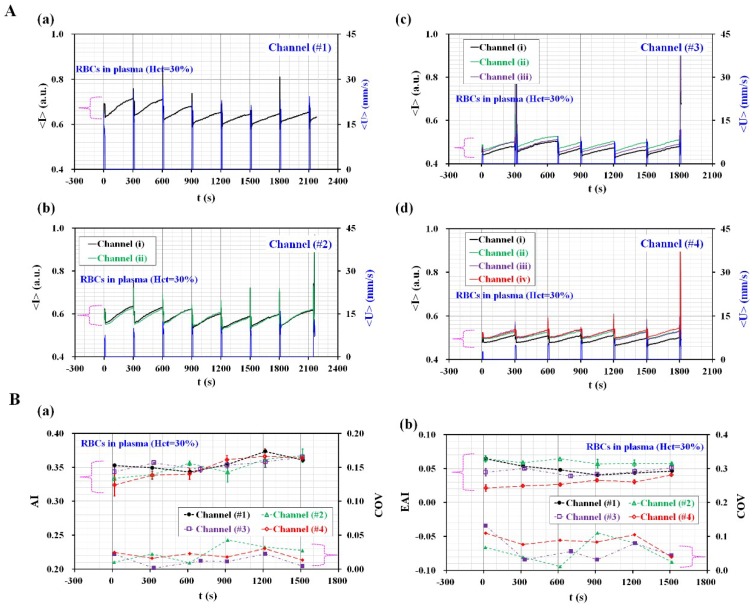
Quantitative evaluation of channel numbers for measuring scattering of two indices (AI and EAI). (**A**) Temporal variations of <*U*> and <*I*> with respect to channel number (*n*) ((**A-a**) *n* = 1, (**A-b**) *n* = 2, (**A-c**) *n* = 3, and (**A-d**) *n* = 4). (**B**) Temporal variations of AI and EAI with respect to channel number. (**B-a**) Temporal variation of AI and, coefficient of variation (COV) with respect to channel number. (**B-b**) Temporal variations of EAI and COV with respect to channel number.

To find out the effect of the channel number on measurement of RBCs aggregation and ESR, the variations of RBC aggregation and ESR (i.e., AI and EAI) were evaluated with respect to number of channel (*n*) (*n* = 1,2, 3, and 4). Figure S1 (Supplementary Materials) showed microscopic images for microfluidic device with multiple numbers of channel (*n*) [(a) *n* = 1, (b) *n* = 2, (c) *n* = 3, and (d) *n* = 4]. Blood sample was prepared by adding normal RBCs into plasma. Hematocrit was fixed at 30%. As shown in Figure 5A, temporal variations of <*I*> and <*U*> were simultaneously measured with respect to channel number. As a result, <*I*> and <*U*> were decreased with increasing channel numbers. When removing pinch valve (i.e., open mode) for 10 s, blood velocity tended to decrease with increasing channel number. Furthermore, image intensity of stasis blood flow tended to decrease for rest time of each period (~290 s). In other words, blood velocity for short duration time (~10 s) contributed to changing image intensity of blood. Temporal variations of two indices (AI and EAI) were obtained by analyzing temporal variations of image intensity for each duration of period. As shown in Figure 5B-a, AI does not show significant difference with respect to channel number. To compare with scattering of AI, coefficient of variation (COV) (~standard deviation/mean) was estimated as less than 0.05 from two channels to four channels. However, since minimum value of image intensity (<*I*>_min_) for each period was inserted to calculate EAI, higher number of channels contributed to decreasing EAI. As shown in Figure 5B-b, four channels showed lower values of EAI compared with the rest of channel numbers. In addition, COV of EAI remained within 0.1 from two channels to four channels.

### 3.4. Quantitative Evaluation of the Effect of Hematocrit Variations

**Figure 6:**
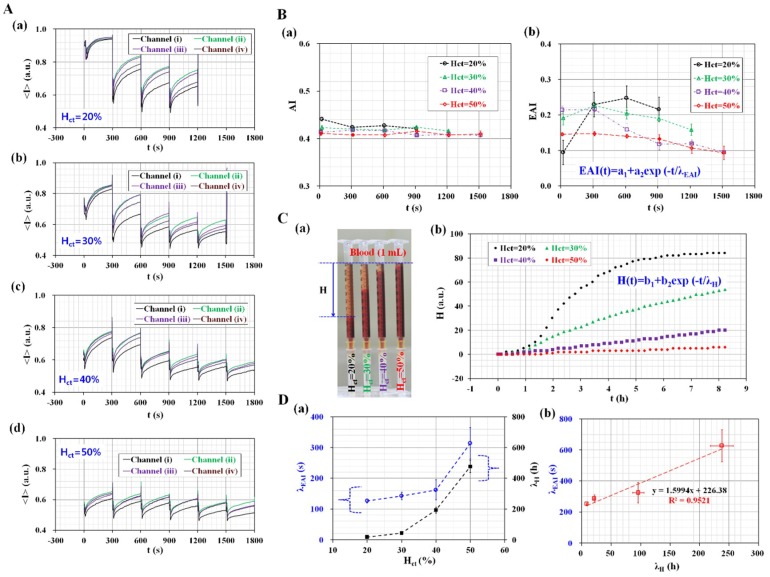
Quantitative evaluation of the effect of hematocrit on the performance of the proposed method. Specifically, RBC aggregation and ESR are quantified using two indices (AI and EAI). Hematocrit (*H*_ct_) (*H*_ct_ = 20%, 30%, 40%, and 50%) was adjusted by adding normal RBCs into autologous plasma. (**A**) Temporal variations of <*I*> with respect to each microfluidic channel and hematocrit (*H*_ct_) ([**A-a**] *H*_ct_ = 20%, [**A-b**] *H*_ct_ = 30%, [**A-c**] *H*_ct_ = 40%, and [**A-d**] *H*_ct_ = 50%). (**B**) Temporal variations of AI and EAI with respect to hematocrit. (**B-a**) Temporal variations of AI with respect to hematocrit. (**B-b**) Temporal variations of EAI with respect to hematocrit. (**C**) ESR measurement using a disposable syringe (~1 mL) as a conventional method. (**C-a**) Snapshot images for quantifying ESR method with respect to hematocrit. Here, *H* represents height in RBCs-depleted layer. (**C-b**) Temporal variations of *H* with respect to hematocrit. (**D**) Quantitative comparison between conventional method and proposed method. (**D-a**) Variations of *λ*_EAI_ and *λ*_H_ with respect to hematocrit (*H*_ct_). (**D-b**) Linear relationship between *λ*_EAI_ and *λ*_H_.

In order to evaluate the effect of hematocrit on the RBC aggregation under continuous ESR, the four different hematocrits (*H*_ct_) (*H*_ct_ = 20%, 30%, 40%, and 50%) were prepared by adding normal RBCs into autologous plasma. RBC aggregation and ESR were quantified using AI and EAI, respectively. Figure 6A shows temporal variations of <*I*> with respect to each microfluidic channel and hematocrit level (*H*_ct_) ([a] *H*_ct_ = 20%, [b] *H*_ct_ = 30%, [c] *H*_ct_ = 40%, and [d] *H*_ct_ = 50%). Image intensity (<*I*>) varied over time with respect to each microfluidic channel and hematocrit. With respect to blood with 20% hematocrit, all RBCs were passed from a pipette tip to outlet port after 1200 s, and thus, variations in image intensity for four microfluidic channels were obtained up to 1200 s. With respect to blood with 30% hematocrit, variations of image intensity were obtained up to 1500 s. However, the other two blood types (*H*_ct_ = 40% and 50%) exhibited a lower value of ESR in a pipette tip, and thus, RBCs continued to exist in the microfluidic channels. Variations in image intensity were obtained up to 1800 s. Blood with lower hematocrit exhibited a higher value of *S*_A_ and *S*_C_ when compared with blood with higher hematocrit. This result indicated that blood sample with lower hematocrit exhibited higher values of ESR and RBC aggregation. Temporal variations of <*I*> were used for four microfluidic channels to obtain temporal variations of AI and EAI with respect to hematocrit. RBCs aggregation was quantified using temporal variations of image intensity from stasis blood flow (*t* = 0). The effect of shear rate on RBCs aggregation measurement was negligible (i.e., $\dot{\gamma }$ = 0). As shown in Figure 6A, since temporal variations of image intensity were obtained by analyzing microscopic images captured at stasis blood flows, RBCs aggregation only contributed to varying image intensity of blood sample in each microfluidic channel. As shown in Figure 6B-a, AI did not display significant differences with respect to hematocrit (*H*_ct_) and measurement time (*t*). Additionally, blood with lower hematocrit exhibited a higher value of AI when compared with blood with higher hematocrit. Furthermore, AI remained constant over time. Conversely, temporal variations of EAI were obtained with respect to hematocrit to include the effect of ESR. As shown in Figure 6B-b, EAI shows a significant difference with respect to hematocrit when compared with AI. Therefore, blood with 20% hematocrit exhibited a higher value of EAI when compared with other blood. Moreover, blood with 40–50% hematocrit exhibited a similar value to EAI after 900 s. The result indicated that EAI was more effective at evaluating the effect of hematocrit on the RBC aggregation when compared with that of AI, because EAI included the effect of ESR in the pipette tip.

As quantitative comparison, the effect of hematocrit on ESR measurement was quantified by using a modified conventional method [15,17]. Here, hematocrit of blood (*H*_ct_) were adjusted to *H*_ct_ = 20%, 30%, 40%, and 50% by adding normal RBCs into autologous plasma. After installing four disposable syringes vertically in base plate, blood with different hematocrit (~1 mL) was dropped into each syringe with a pipette as shown in Figure 6C-a. At intervals of 30 s, consecutive images were captured with a digital camera (D700, Nikon, Tokyo, Japan). The ESR was then quantified by measuring the height of RBCs-depleted layer (i.e., *H*) over time. As shown in Figure 6C-b, temporal variations of *H* were obtained by varying hematocrit. After then, to quantify relationship between proposed method (i.e., EAI) and conventional method (i.e., *H*), simple regression formula for each method was used as EAI = *a*_1_ + *a*_2_ exp (−*t*/*λ*_EAI_) and *H*(*t*) = *b*_1_ + *b*_2_ exp(−*t*/*λ*_H_), respectively. Here, *λ*_EAI_ and *λ*_H_ denote corresponding time constants for proposed method and conventional method. By conducting nonlinear regression analysis with a commercial software (Matlab, Mathworks, Natick, MA, USA), variations of *λ*_EAI_ and *λ*_H_ were obtained with increasing hematocrit. As shown in Figure 6D-a, both time constants tend to increase gradually with respect to hematocrit. In other words, ESR is saturated within a short time for lower hematocrit, compared with higher hematocrit. In addition, EAI shows very similar trend with respect to hematocrit. To find out relationship between two time constants (*λ*_EAI_ and *λ*_H_), linear regression analysis was conducted using EXCEL program (Microsoft^TM^, Natick, MA, USA). As shown in Figure 6D-b, coefficient of *R*^2^ shows sufficient high value of *R*^2^ = 0.9521. From this result, both methods have significantly linear relation. Furthermore, it leads to conclusion that EAI as newly suggested index can be effectively employed to monitor variation of ESR through quantifying image intensity of blood flowing in microfluidic channels.

### 3.5. Quantitative Evaluation of RBC Aggregation under Continuous for Homogeneous Aggregated RBCs

A performance demonstration involved applying the proposed method to measure RBC aggregation under continuous ESR for homogeneous aggregated RBCs. In order to elevate RBC aggregation for normal RBCs, three different concentrations of dextran solution (*C*_dextran_) (*C*_dextran_ = 5 mg/mL, 15 mg/mL, and 20 mg/mL) were prepared by adding dextran into 1× PBS solution. Subsequently, hematocrit of the blood sample was adjusted to 30% by adding normal RBCs into three different concentrations of the dextran solution. Additionally, a control blood sample was prepared by adding normal RBCs into 1× PBS solution (*C*_dextran_ = 0) to exclude the effect of RBC aggregation.

**Figure 7:**
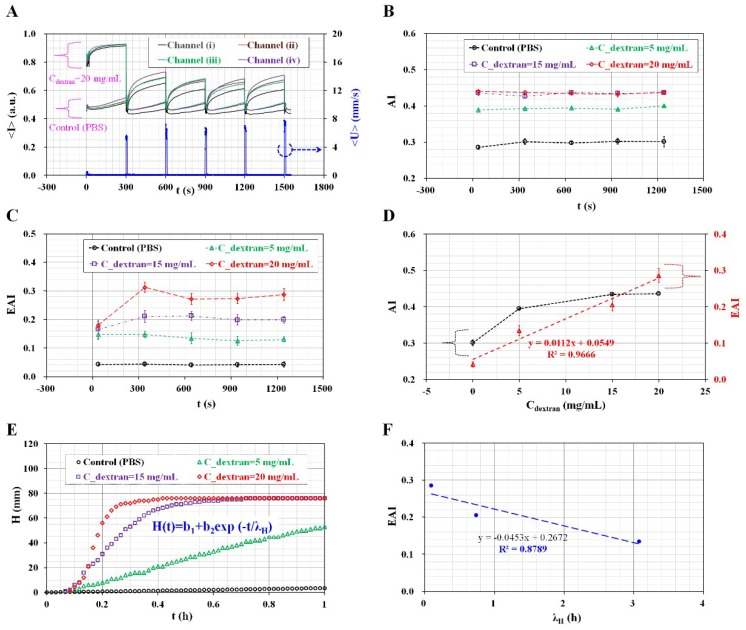
Quantitative evaluations of RBC aggregation (i.e., AI) and ESR (i.e., EAI) with the proposed method and the modified conventional ESR method. To elevate RBC aggregation and ESR, three concentrations of dextran solution (*C*_dextran_) (*C*_dextran_ = 5 mg/mL, 15 mg/mL, and 20 mg/mL) were prepared by adding dextran into a PBS solution. Hematocrit is adjusted to 30% by adding normal RBCs into three different concentrations of the dextran solution. (**A**) Temporal variations of <*I*> and <*U*> for two different blood samples (*C*_dextran_ = 0, 20 mg/mL). (**B**) Temporal variations of AI with increasing concentrations of the dextran solution. (**C**) Temporal variations of EAI with increasing concentrations of the dextran solution. (**D**) Variations of AI and EAI with respect to the concentration of the dextran solution. (**E**) Temporal variations of *H* with respect to specific concentrations of dextran solution. Here, *H* denotes height of RBCs-depleted layer in a disposable syringe. (**F**) Linear relationship between proposed method (i.e., EAI) and the modified conventional ESR method (i.e., *λ*_H_).

Figure 7A shows temporal variations of <*I*> and <*U*> for two different bloods prepared by adding normal RBCs into 20 mg/mL dextran solution and PBS solution, respectively. The tube opened shortly at an interval of 300 s, and thus, an average of blood velocity (<*U*>) was measured as a periodic pulse-like shape. Image intensity (<*I*>) tended to immediately increase over time when the pinch valve clamps the tube (i.e., in the close mode). A blood sample with a higher concentration of the dextran solution (*C*_dextran_ = 20 mg/mL) exhibited larger variations in image intensity (i.e., *S*_A_), when compared with that of the control blood sample (C_dextran_ = 0). For two blood samples, a minimum value of <*I*> (<*I*>_min_) exhibited a significant decrease at *t* = 300 s. Following this, <*I*>_min_ remained constant with an elapse of time. This result indicated that ESR occurs dominantly within 300 s. After 300 s, ESR remained constant with time elapses.

In order to quantify RBC aggregation under continuous ESR, two indices (AI and EAI) were calculated using temporal variations of <*I*> for each microfluidic channel. As shown in Figure 7B, the temporal variations of AI were obtained with respect to concentrations of the dextran solution. The control blood sample (i.e., normal RBC in PBS suspension) showed a lower value of AI = 0.30 ± 0.01 when compared with aggregated blood sample (i.e., normal RBCs in plasma), as shown in Figure 6B-a. Hence, the AI gives higher value of reference, since control blood sample was prepared to exclude RBC aggregation. Thus, it was estimated that lower hematocrit (*H*_ct_ = 30%) contributed to a higher value of AI as a reference value. Additionally, the blood sample exhibited a higher value of AI with increases in the concentration of the dextran solution. However, AI did not indicate significant differences for blood samples, with concentrations of the dextran solution ranging from *C*_dextran_ = 15 mg/mL to *C*_dextran_ = 20 mg/mL.

As shown in Figure 7C, temporal variation of EAI was calculated using temporal variations of image intensity (<*I*>) with respect to concentrations of the dextran solution. As a result, EAI remained constant after 2 cycles. Since each cycle is continued for five min., the proposed method requires at least 10 min. The proposed method does remove necessity of repetitive test as significant advantage. The control blood sample exhibited a significantly lower value of EAI (i.e., EAI = 0.04 ± 0.01) as a reference value when compared with that of the AI. Overall variations in EAI are significantly higher than those of AI within a specific dextran solution. Furthermore, EAI exhibited a significant difference at specific dextran concentrations ranging from *C*_dextran_ = 15 mg/mL to *C*_dextran_ = 20 mg/mL.

Finally, as shown in Figure 7D, variations of AI and EAI were displayed with respect to a specific dextran solution. The linear regression analysis indicated a high value of *R*^2^ (i.e., *R*^2^ = 0.9666), and thus, EAI linearly varied within a specific concentration of dextran solution. However, AI varied gradually below 15 mg/mL dextran solution. Furthermore, AI approached saturation above 15 mg/mL dextran solution. According to most previous methods, a blood sample was directly dropped into an inlet port of the microfluidic device. Since hematocrit of the blood sample remained constant in a microfluidic channel, the previous methods did not require to consider the effect of continuous ESR in the reservoir on the RBCs aggregation. In other words, the previous method did not consider the effect of hematocrit variations on the syllectogram (i.e., *S*_C_ = 0). Based on previous methods [2,3], the RBC aggregation was quantified by calculating AI as AI = *S*_A_/(*S*_A_ + *S*_B_). However, in this study, the proposed method involved simultaneously measuring RBC aggregation and ESR in conical pipette tip. The continuous ESR caused to increase hematocrit of blood supplied into a microfluidic channel. According to temporal variations of <*I*>, minimum value of <*I*> (i.e., <*I*>_min_) decreased due to increases in hematocrit. In order to quantify RBC aggregation due to ESR in the conical pipette tip, it was necessary to simultaneously consider variations in <*I*> due to RBC aggregation (i.e., *S*_A_) and <*I*>_min_ due to ESR (i.e., *S*_C_). Thus, EAI is evaluated as EAI = *S*_A_/*S*_C_.

### 3.6. Quantitative Comparison between the Proposed Method and the Modified Conventional ESR Method

To compare with the experimental results obtained by the proposed method, blood was prepared by adding normal RBCs into a specific concentration of dextran solution (*C*_dextran_) (*C*_dextran_ = 0, 5 mg/mL, 15 mg/mL, and 20 mg/mL). Here, *C*_dextran_ = 0 denotes 1× PBS solution. Thereafter, 1 mL blood was dropped into a disposable syringe (1 mL, BD Science, Singapore) as a modified conventional method [15,17]. Figure 7E shows temporal variations of H by varying various concentrations of dextran solution. As a result, dextran solution contributes to increasing H significantly, compared with control blood. To quantify variations of ESR, simple regression expression was suggested as *H*(*t*) = *b*_1_ + *b*_2_ exp (−*t*/*λ*_H_). Then, time constant (i.e., *λ*_H_) was obtained by conducting nonlinear regression analysis with commercial software. Figure 7F represents the quantitative comparison between proposed method (i.e., EAI) and the conventional method (i.e., *λ*_H_). To verify the relationship between the proposed method and conventional method, a linear regression analysis was conducted with EXCEL program (Microsoft^TM^, Natick, MA, USA). Since the coefficient of *R*^2^ has higher value of *R*^2^ = 0.8789, the EAI obtained by the proposed method can give comparable value of ESR, compared with the conventional method. As shown in Figure 6B-b, EAI of normal blood (*H*_ct_ = 30%) was varied from 0.16 to 0.22. According to Figure 7D, EAI was increased linearly with increasing concentration of dextran solution. Blood with lower concentration of dextran solution (*C*_dextran_ = 5 mg/mL) had EAI = 0.134 ± 0.014. In other words, 5 mg/mL dextran solution played a similar ESR behavior, compared with plasma solution. Blood prepared with higher concentration of dextran solution (*C*_dextran_ = 20 mg/mL) had EAI = 0.286 ± 0.019. From this result, maximum concentration of dextran solution (20 mg/mL) contributed to increasing EAI twice, compared with normal blood. According to previous study [22], using streptozotocin-induced rats, variations of ESR were measured by varying duration of diabetes (*D*_Diabetes_). Compared with control, ESR was increased over twice. From this results, EAI obtained by the proposed method can be effectively used to detect variations of ESR or RBCs aggregation.

From this experimental demonstration, it is found that the suggested method is able to quantify variations of RBC aggregation under continuous ESR. In other words, EAI is more effective when compared with AI. Furthermore, the method provides multiple data of RBC aggregation and ESR through a single experiment. The rheological property varied continuously, and thus it is very effective at obtaining several data points without repetitive tests. Compared with the previous methods, this proposed method had some merits including fabrication, multiple channels, and quantitative comparison of ESR value. First, the use of a liner cut from an adhesive sheet to form the master mold was newly suggested to simplify the fabrication process, compared to MEMS fabrication. Since RBC aggregation is quantified under stasis blood flows or lower shear rates, it is not imperative that each microfluidic channel should have uniform sizes for consistent blood flows. Thus, this method can remove MEMS fabrications, which require high cost and technical expertise. Second, when RBC aggregation varied over time, the repetitive test caused to increase scattering of aggregation index largely. Thus, multiple measurement of RBC aggregation was required to avoid large scattering due to repetitive test conducted for long time. At last, ESR relationship between modified conventional method and proposed method was obtained by conducting regression analysis technique. As a result, the EAI obtained by the proposed method gave comparable value of ESR, compared with the modified conventional ESR method.

## 4. Conclusions

In this study, a simple measurement technique of RBC aggregation under continuous ESR was demonstrated by sucking blood from a conical pipette tip into parallel microfluidic channels and quantifying image intensity, especially throughout single experiment. Two indices (AI and EAI) were suggested to quantify variations of RBC aggregation due to ESR in the conical pipette tip. First, when clamping the fluidic tube with the pinch valve, blood flow in each microfluidic channel was stopped shortly within 0.2 s. RBCs aggregation was measured immediately after clamping the tube with a pinch valve (i.e., close mode). Additionally, the compliance effect of the fluidic system had a negligible effect on transient blood flows. In addition, AI and EAI remained constant without respect to flow rate (*Q*) (*Q* = 0.5 mL/h, 1 mL/h, and 2 mL/h). From this result, RBC aggregation and ESR were measured immediately by clamping the tube with a pinch valve, at the constant flow rate of 2 mL/h. Second, the proposed method was applied to measure the effect of hematocrit on the RBC aggregation under continuous ESR. AI remained over time with respect to hematocrit. However, EAI showed a significant difference with respect to hematocrit and measurement time. Compared with AI, EAI was more effective for evaluating continuous ESR in the conical pipette. After that, to quantify the relationship between the proposed method (i.e., EAI) and the modified conventional ESR method (i.e., *H*) with respect to hematocrit level, time constants (*λ*_EAI_, *λ*_H_) were obtained by conducting regression analysis with simple regression formula [i.e., EAI = *a*_1_ + *a*_2_ exp (−*t*/*λ*_EAI_), H = *b*_1_ + *b*_2_ exp (−*t*/*λ*_H_)]. For two time constants (*λ*_EAI_, *λ*_H_) with respect to hematocrit, linear regression analysis indicated that the coefficient of *R*^2^ showed a sufficient high value of *R*^2^ = 0.9521. Thus, EAI can be effectively employed to measure ESR in the reservoir, compared with a modified conventional ESR method. The proposed method was finally applied to evaluate RBC aggregation (AI) and ESR (EAI) for homogeneous aggregated RBCs. As a result, EAI and AI were gradually increased by varying concentration of dextran solution ranging from *C*_dextran_ = 5 mg/mL to *C*_dextran_ = 20 mg/mL. To evaluate the relationship between the proposed method and a modified conventional ESR method, EAI and *λ*_H_ were obtained at specific concentrations of dextran solution. Since regression analysis showed higher value of *R*^2^ = 0.8789, the EAI obtained by the proposed method gave comparable value of ESR, compared with the modified conventional ESR method. These experimental demonstrations indicated that the proposed method simultaneously measures RBC aggregation and ESR by using two indices (AI and EAI). Moreover, the method provides multiple data of RBC aggregation and ESR through a single experiment. Future tests will involve employing the proposed method to evaluate biophysical properties of bloods collected from cardiovascular diseases.

## Supplementary Materials

The following are available online at http://www.mdpi.com/2072-666X/9/7/318/s1: Figure S1: Microscopic images for showing a microfluidic device with parallel microfluidic channel (*n*) [(a) *n* = 1, (b) *n* = 2, (c) *n* = 3, and (d) *n* = 4].

## References

[1] Lanotte L., Mauer J., Mendez S., Fedosov D.A., Fromental J.-M., Claveria V., Nicoud F., Gompper G., Abkarian M. (2016). Red cells’ dynamic morphologies govern blood shear thinning under microcirculatory flow conditions. Proc. Natl. Acad. Sci. USA.

[2] Baskurt O.K., Meiselman H.J. (2010). Time course of electrical impedance during red blood cell aggregation in a glass tube: Comparison with light transmittance. IEEE Trans. Biomed. Eng..

[3] Isiksacan Z., Erel O., Elbuken C. (2016). A portable microfluidic system for rapid measurement of the erythrocyte sedimentation rate. Lab Chip.

[4] Popel A.S., Johnson P.C. (2005). Microcirculation and hemorheology. Annu. Rev. Fluid Mech..

[5] Bishop J.J., Popel A.S., Intaglietta M., Johnson P.C. (2001). Rheological effects of red blood cell aggregation in the venous network: A review of recent studies. Biorheology.

[6] Yayan J. (2012). Erythrocyte sedimentation rate as a marker for coronary heart disease. Vasc. Health Risk Manag..

[7] Bochen K., Krasowska A., Milaniuk S., Kulczyńska M., Prystupa A., Dzida G. (2011). Erythrocyte sedimentation rate-an old marker with new applications. JPCCR.

[8] Piva E., Pajola R., Temporin V., Plebani M. (2007). A new turbidimetric standard to improve the quality assurance of the erythrocyte sedimentation rate measurement. Clin. Biochem..

[9] Larsson A., Hansson L.-O. (2005). Inflammatory activity: Capillary electrophoresis provides more information than erythrocyte sedimentation rate. Upsala J. Med. Sci..

[10] Fabry T.L. (1987). Mechanism of erythrocyte aggregation and sedimentation. Blood.

[11] Cha C.-H., Park C.-J., Cha Y.J., Kim H.K., Kim D.H., Bae J.H., Jung J.-S., Jang S., Chi H.-S., Lee D.S. (2009). Erythrocyte sedimentation rate measurements by test 1 better reflect inflammation than do those by the Westergren method in patients with malignancy, autoimmune disease, or infection. Am. J. Clin. Pathol..

[12] Plebani M., Toni S.D., Sanzari M.C., Bernardi D., Stockreiter E. (1998). A New method for mMeasuring the erythrocyte sedimentation rate. Am. J. Clin. Pathol..

[13] Kaliviotis E., Sherwood M., Balabani S. (2017). Partitioning of red blood cell aggregates in bifurcating microscale flows. Sci. Rep..

[14] Kang Y.J. (2016). Continuous and simultaneous measurement of the biophysical properties of blood in a microfluidic environment. Analyst.

[15] Kang Y.J., Ha Y.-R., Lee S.-J. (2014). Microfluidic-based measurement of erythrocyte sedimentation rate for biophysical assessment of blood in an in vivo malaria-infected mouse. Biomicrofluidics.

[16] Shin S., Hou J.X., Suh J.-S. (2007). Measurement of cell aggregation characteristics by analysis of laser-backscattering in a microfluidic rheometry. Korea-Aust. Rheol. J..

[17] Yeom E., Lee S.J. (2015). Microfluidic-based speckle analysis for sensitive measurement of erythrocyte aggregation: A comparison of four methods for detection of elevated erythrocyte aggregation in diabetic rat blood. Biomicrofluidics.

[18] Zhbanov A., Yang S. (2015). Effects of aggregation on blood sedimentation and conductivity. PLoS ONE.

[19] Kang Y.J. (2017). Microfluidic-based measurement method of red blood cell aggregation under hematocrit variations. Sensors.

[20] Uyuklu M., Cengiz M., Ulker P., Hever T., Tripette J., Connes P., Nemeth N., Meiselman H.J., Baskurt O.K. (2009). Effects of storage duration and temperature of human blood on red cell deformability and aggregation. Clin. Hemorheol. Microcirc..

[21] Lim H.-J., Nam J.-H., Lee B.-K., Suh J.-S., Shin S. (2011). Alteration of red blood cell aggregation during blood storage. Korea-Aust. Rheol. J..

[22] Berezina T.L., Zaets S.B., Morgan C., Spillert C.R., Kamiyama M., Spolarics Z., Deitch E.A., Machiedo G.W. (2002). Influence of storage on red blood cell rheological properties. J. Surg. Res..

[23] Bartholomeusz D.A., Boutté R., Andrade J.D. (2005). Xurography: Rapid prototyping of microstructures using a cutting plotter. J. Microelectromech. Syst..

[24] Faustino V., Catarino S.O., Lima R., Minas G. (2016). Biomedical microfluidic devices by using low-cost fabrication techniques: A review. J. Biomech..

[25] Pinto E., Faustino V., Rodrigues R.O., Pinho D., Garcia V., Miranda J.M., Lima R. (2015). A rapid and low-cost nonlithographic method to fabricate biomedical microdevices for blood flow analysis. Micromachines.

[26] Islam M., Natu R., Martinez-Duarte R. (2015). A study on the limits and advantages of using a desktop cutter plotter to fabricate microfluidic networks. Microfluid. Nanofluid..

[27] Bento D., Sousa L., Yaginuma T., Garcia V., Lima R., Miranda J.M. (2017). Microbubble moving in blood flow in microchannels: Effect on the cell-free layer and cell local concentration. Biomed. Microdevices.

[28] Yeom E., Kim H.M., Park J.H., Choi W., Doh J., Lee S.J. (2017). Microfluidic system for monitoring temporal variations of hemorheological properties and platelet adhesion in LPS-injected rats. Sci. Rep..

