# Supplementary material for: Multiple and Periodic Measurement of RBC Aggregation and ESR in Parallel Microfluidic Channels under On-Off Blood Flow Control
Source: Micromachines (Basel). 2018 Jun 24;9(7):318. doi: 10.3390/mi9070318 (PMC6082273; doi:10.3390/mi9070318)
Supplement: Supplementary file 1 [file micromachines-09-00318-s001.pdf]

micromachines

Article

Multiple and Periodic Measurement of RBC Aggregation and ESR in Parallel Microfluidic Channels under On-Off Blood Flow Control

Yang Jun Kang [1,*] and Byung Jun Kim [2]

[1] Department of Mechanical Engineering, Chosun University, 309 Pilmun-daero, Dong-gu, Gwangju 61452, Korea

[2] Department of Biomedical Science and Engineering, Gwangju Institute of Science and Technology (GIST), Gwangju 61005, Korea; gene392@gist.ac.kr

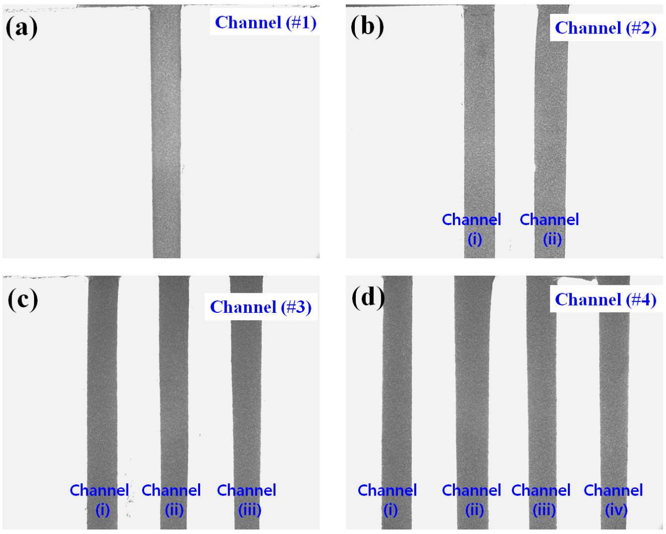

Figure. S1 Microscopic images for showing a microfluidic device with parallel microfluidic channel (*n*) [**(a)** n=1, **(b)** *n*=2, **(c)** *n*=3, and **(d)** *n*=4].
